# The human foot and heel–sole–toe walking strategy: a mechanism enabling an inverted pendular gait with low isometric muscle force?

**DOI:** 10.1098/rsif.2012.0179

**Published:** 2012-05-09

**Authors:** J. R. Usherwood, A. J. Channon, J. P. Myatt, J. W. Rankin, T. Y. Hubel

**Affiliations:** Structure and Motion Laboratory, The Royal Veterinary College, North Mymms, Hatfield, Herts AL9 7TA, UK

**Keywords:** walk, foot, inverted pendulum, plantigrade, heel

## Abstract

Mechanically, the most economical gait for slow bipedal locomotion requires walking as an ‘inverted pendulum’, with: I, an impulsive, energy-dissipating leg compression at the beginning of stance; II, a stiff-limbed vault; and III, an impulsive, powering push-off at the end of stance. The characteristic ‘M’-shaped vertical ground reaction forces of walking in humans reflect this impulse–vault–impulse strategy. Humans achieve this gait by dissipating energy during the heel-to-sole transition in early stance, approximately stiff-limbed, flat-footed vaulting over midstance and ankle plantarflexion (powering the toes down) in late stance. Here, we show that the ‘M’-shaped walking ground reaction force profile does not require the plantigrade human foot or heel–sole–toe stance; it is maintained in tip–toe and high-heel walking as well as in ostriches. However, the unusual, stiff, human foot structure—with ground-contacting heel behind ankle and toes in front—enables both *mechanically economical* inverted pendular walking and *physiologically economical* muscle loading, by producing extreme changes in mechanical advantage between muscles and ground reaction forces. With a human foot, and heel–sole–toe strategy during stance, the shin muscles that dissipate energy, or calf muscles that power the push-off, need not be loaded at all—largely avoiding the ‘cost of muscle force’—during the passive vaulting phase.

## 1. Introduction

The morphology and action of the human foot with—during walking—a grounded ‘heel’ behind a relatively distal ankle joint loaded early in stance, and ‘toes’ pushing off at the end of stance (i.e. a the heel–sole–toe stance or ‘plantigrade’ foot) is very unusual outside the hominoidea (apes including humans) [1–4]. It is absent in the majority of cursors, whether bipedal (e.g. ostrich, emu, etc.) or quadrupedal. While humans have been considered by some as specialized endurance runners [5,6], the role—even the presence—of the heelstrike in natural running is controversial [7]. We therefore consider the potential benefits of the peculiar human foot and heel–sole–toe walking strategy from the perspective of the mechanics and physiology of walking.

### 1.1. Background 1. The inverted pendulum

**Figure 1.**
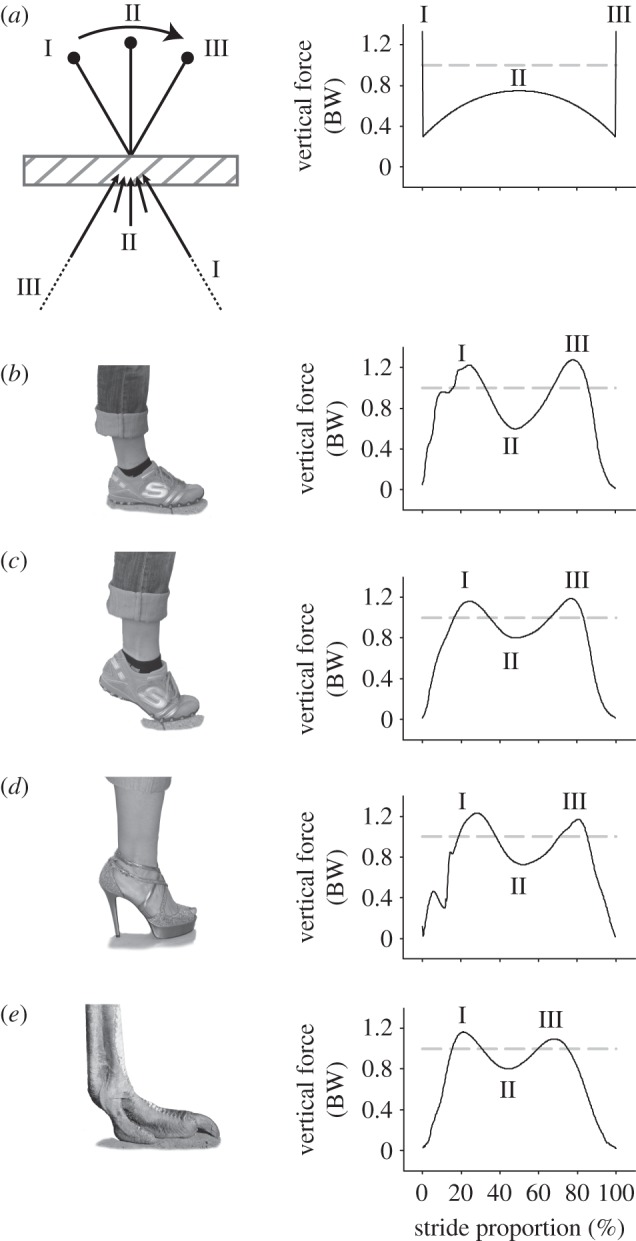
The ideal vaulting inverted pendulum (*a*) shows three phases: I, impulsive energy dissipation in early stance; II, stiff-limbed, passive vaulting; and III, impulsive energy input in late stance. Walking with such a gait requires very high forces; more smoothed, but identifiably related, vertical force profiles are observed in walking humans (*b*). This is maintained even if the usual mechanism for energy loss (ankle plantarfexion in early stance) is removed due to walking on tip–toes (*c*), or energy input (platarflexion late in stance) is limited owing to walking on very high heels (*d*). Further, ostriches, despite walking with no functional ‘heel’, also display the characteristic M-shaped ground reaction force (*e*). Single-limb force measurements are for representative stances during walking at intermediate speeds. See electronic supplementary material, Methods for details.

**Figure 2.**
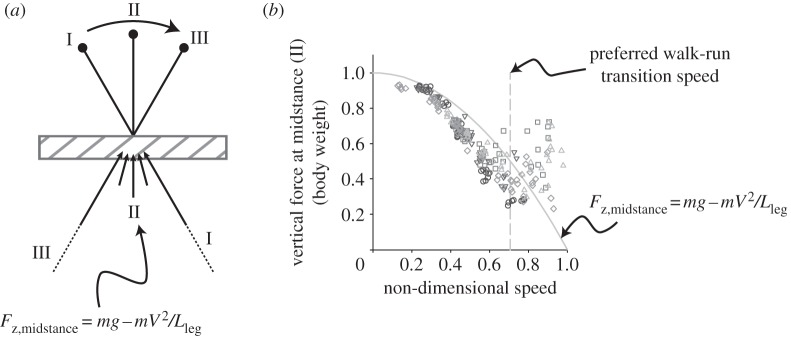
The vertical ground reaction forces at midstance (II) for a stiff-limbed vaulter (*a*), predicted because of the combination of gravitational and centripetal acceleration requirements, (*b*, grey curve) observed for five subjects (symbols) walking over a range of speeds up to, and exceeding, their preferred walk–run transition speeds. Midstance forces are broadly consistent with stiff-limbed vaulting up to the preferred walk–run transition speed. Speed is made non-dimensional—relating to ‘Froude number’—by dividing by the square root of gravity and leg length.

Up-and-down motions have long been identified as characteristic of walking (e.g. Aristotle ‘On the gait of animals’). With the advent of forceplate measurements, the inverted pendulum has become both a description and proposed mechanism of walking, at least during the vaulting phase. This vaulting action (figure 1*a*) is recognized as an energetically effective means of allowing forward motion [8]. Further, modelling walking as an inverted pendulum is successfully predictive, accounting for the differing relative maximum walk speeds in birds and humans [9–11] owing to differing relative step frequencies, and for the vertical force at midstance in walking humans (figure 2, following Alexander [9]).

More recently, consideration has been paid to the energetic consequences of the transition between each vault [12]. Theoretical collisional analysis demonstrates that some means of powering are less costly than others [13,14]. Further, computer optimization of point-mass models finds that stiff-limbed vaulting with the powering and dissipative impulses predicted (figure 1*a*) from the purely collisional approach is indeed energetically optimal at walking speeds [15].

The mechanisms by which these powering and dissipative impulses are applied in humans—predominantly ankle extension (plantarflexion) powered by the calf muscles (soleus and gastrocnemius) at the end of stance, and ankle extension opposed by the shin muscles (predominantly tibialis anterior, TA) at the beginning of stance—has been related to traditional inverse dynamics measurements [16]. Results from computer simulations using more detailed musculoskeletal models are also consistent with these mechanisms. TA activity continues beyond swing into early stance where its fibres are actively stretching [17]. Both main calf muscles increase in activity to generate positive mechanical work during late stance [18] that greatly contributes to the vertical ground reaction force, accelerating the centre of mass upwards in this period [19,20]. Such accounts are persuasive in that they demonstrate the mechanism by which humans usually achieve walking broadly consistent with theoretically economical gaits. However, they do not answer why the leg should telescope at the ankle, which is neither a theoretical requirement [21] nor consistent with the maintenance of the M-shaped vertical ground reaction force profile in tip–toe, high-heel and ostrich walking (figure 1*b–e* and see also electronic supplementary material, Methods). Here, we take the theoretical ideal of inverted pendular walking with impulsive (high force, short duration) telescope-powering and loss, and now include the biological peculiarity of a ‘cost of muscle force’, to account for the plantigrade foot of humans, suggesting this structure and the heel–sole–toe walking strategy to be a specialization for efficient walking.

### 1.2. Background 2. The ‘cost of muscle force’ and mechanical advantage

We argue here that, to account for the stiff plantigrade human foot (as distinct from other, more compliant, ape feet), with heel behind ankle and toe in front, and the heel–sole–toe walking strategy, requires an appreciation of *both* the energetically optimal powering/support strategy of vaulting, impulsive inverted pendular walking *and* the cost of muscle force—which may be reduced by simple mechanisms that alter the mechanical advantage [22] between the muscle and the ground reaction force. An energetic cost to isometric (constant-length) force on the actuator may be counterintuitive—it is often near-negligible in engineered systems—but is quickly apparent if you attempt to stand with flexed knees. It has been shown to increase the cost of locomotion by 50 per cent in humans walking with a chimpanzee-like bent-hip bent-knee (BHBK) gait [23], and is highly relevant to the metabolic cost of locomotion, both in ‘normal’, competent cursors [24,25] and in the BHBK chimpanzee [26]. In general, human walking is achieved with relatively high muscle mechanical advantages (compared with running), allowing ground reaction forces to be supported with relatively small muscle forces [27,28].

## 2. The phases of stance

Here, we treat stance as being composed of three discrete events. This is clearly somewhat reductionist; in reality, the transition between phases is smooth. Our simplification provides an alternative reduction of walking to a previous attempt to account for human foot structure and the translation of the centre of pressure during stance, which supposes that ‘the plantigrade human foot rolls over the ground during each walking step, roughly analogous to a wheel’ [29]

### 2.1. Very early stance—I

Walking economically with an inverted pendulum gait requires a loss in mechanical energy of the centre of mass in early stance. Although under certain circumstances (running or hopping in animals with long tendons) a large proportion of such mechanical energy loss can be stored and returned via elastic structures (and spring-mass models can achieve something close to walking gaits [30]), this does not appear to be the case in human walking; in humans, the tendon most typically associated with elastic energy storage (the Achilles) is not intensely loaded during early stance, when mechanical energy is being lost. Thus, the energy must be dissipated. While some energy is certainly dissipated owing to deflections of soft tissues throughout the body [16], muscles that dorsiflex the ankle (e.g. fibulari, TA) could also act as effective energy dissipators by resisting plantarflexion (i.e. eccentric contraction). In particular, the TA has received extensive focus across research areas, providing much evidence suggesting the muscle provides this function. For example, ape species demonstrating BHBK gaits possess less voluminous TAs than those that walk with an upright stiff-limbed gait (*Pan* (2.4% of body mass) as well as *Hylobates* (2.1%) relative to *Homo* (4%) and *Pongo* (3.2%) [31]). Experimental electromyographic (EMG) data consistently have two major bursts of activity for this muscle [32,33], with the largest burst occurring just after heel-strike followed by a second bust shortly after toe-off; and TA EMG activity is reduced during tip–toe walking in humans [28]. Although relating muscle force to EMG data is tenuous, the relatively larger burst of EMG activity during early stance suggests that the TA probably functions more to dissipate energy through eccentric contraction than to help provide toe-lift during early swing. Studies using detailed human musculoskeletal walking simulations also suggest the TA acts primarily as an energy absorber [34,35]. These studies show that, relative to normal walking, the TA increases its negative contribution to the vertical impulse (i.e. energy absorption) under added mass and weight conditions, and, at the preferred walking speed, total mechanical work generated during swing is small. Of course, the TA does act to dorsiflect the foot, facilitating toe clearance, during the swing phase. But this requires relatively low loads and little positive work and, at least in evolutionary terms, alternative toe-clearing strategies can be imagined; perhaps this dorsiflection during swing should be viewed as merely preparation for the dissipitive, high-load plantarflexion in early stance.

**Figure 3.**
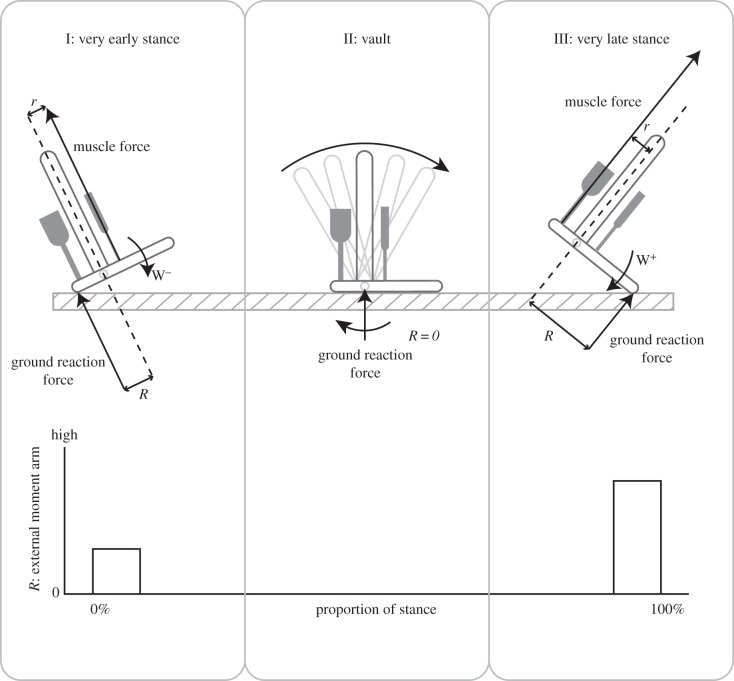
Idealized phases of stance using a human-style foot and heel–sole–toe strategy in walking. In very early stance (I) the heel is loaded, resulting in an external moment arm *R* about the ankle, allowing the energy dissipation *W*^−^ from the ‘shin’ muscles required to initiate the inverted pendular vault. During the vault (II), the sole contacts the ground, and the ground reaction force passes through the ankle, resulting in zero external moment arm, thus avoiding unnecessary and costly loading of either ‘shin’ or ‘calf’ muscles. In very late stance (III), the foot is loaded in front of the ankle, towards the toes, producing an external moment arm, allowing the calf muscles to be loaded during contraction, producing the positive work *W*^+^ required to power inverted pendular walking. The alternative strategy, involving flexion and extension of the knee, results in high muscle loading during the vault phase (electronic supplementary material, figure S1).

If the TA indeed primarily acts as an energy-losing specialist (figure 3I), the muscle's role is fundamentally different from the roles of work generation or force resistance traditionally assigned to muscle [36]. However, there may be advantages to using a specialized muscle for such a role, despite the metabolic cost of ‘negative work’ when performed by muscles: energy lost in the muscle need not be lost in more brittle structures within the body. Muscles, unlike large-strain passive dissipitive structures, are capable of many cycles without creep, and muscle allows a certain degree of control. While energy *can* be lost in stiff structures (e.g. fat pads, bone, etc.), large-deflection energy losses keep forces and the corresponding tendency to lose energy from non-specialized structures low. However, this preferential use of TA to dissipate energy through eccentric contraction during human walking may relate to a tendancy towards muscle soreness, and potentially injury (sometimes, perhaps erroneously, referred to as ‘shin splints’), through walking at high speeds and under high loads. The full implications of a muscle that might, unusually, be specialized for energy loss is exciting, but beyond the scope of this study.

### 2.2. The vault—II

The vault is mechanically passive if the stance limb is maintained at a constant length, and torques are not applied about the centre of mass. While detailed models of human walking show that significant work may, in reality, be performed during the vault at the level of the muscles [37]; at a whole-body level, the stiff-limbed vaulting model of walking is remarkably effective, both accounting for top walking speeds in humans and birds [10,11], and the forces at midstance *F*_z,midstance_. Extending Alexander's 1976 ‘stiff-legged walk’ analysis [9]:
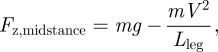
where force is reduced from body weight *mg* owing to the ‘centrifugal force’ of the mass *m* describing an arc of radius leg length *L*_leg_ at a walking speed of *V*. This is the force at the ‘trough’ in the M-shaped ground reaction force curve, and fits empirical measurements of walking (figure 2), at least below the preferred walk–run transition speed.

During the vault, the human foot lies flat on the ground. Thus, the ground reaction force is able to pass close to the ankle (figure 3II)—the external moment arm *R* tends to zero—thus applying little to no loading to either the energy-dissipating shin muscle active during early stance, nor the powering calf muscles active in late stance. The plantigrade foot, therefore, not only allows energy loss and powering as predicted to be energetically optimal at a mechanical level (this could equally well be achieved at the knee—see electronic supplementary material, figure S1), but it also removes the requirement for muscles to be loaded while performing no work.

### 2.3. Very late stance—III

At the end of stance, when an impulse and positive work is required to power the body into the next step, the foot is loaded by the ground reaction force in front of the ankle (towards the toes). This results in an external moment arm (figure 3III), enabling work by the calf—gastrocnemius and soleus—muscles to power the leg extension by pulling on the heel end of the foot, which acts as a lever, stiffened by the ‘longitundinal arch’.

This view of walking provides insight into the role of elasticity for the Achilles tendon (the tendon connecting the gastrocnemius to the back of the foot). It is thought that elasticity in this tendon allows some form of energy saving during walking [38]. Near the end of the vault, the calf muscles increase activity but generate isometric force while allowing their tendons to stretch. This provides an opportunity to prepare for power generation in very late stance while both operating on more favourable regions of their force–velocity curves, and increasing the duration of activity [39,40].

However, there is a limit to the amount of energy that can be usefully stored in the Achilles tendon during inverted pendular walking: tension along the Achilles that results in ankle extension before the end of stance would result in energetically costly deviation from the ideal inverted pendular gait. Further, the load—and thus energy able to be stored—in this tendon before it extends the ankle would, according to inverted pendular mechanics, go down with speed and step length [9,10]; indeed, at top walking speeds, the compression force along the leg—and hence the ground reaction force available to load the tendon—theoretically, falls toward zero. Consistent with this prediction from the inverted pendular view of walking, this phenomenon is also consistent with findings from the vastly more sophisticated and complete walking model of Neptune *et al*. [18], which finds that muscle fibre work increases relative to tendon work in walking speeds from 1.20 to 2.0 m s^−1^.

Therefore, the benefits in terms of physiological muscle efficiency of an elastic tendon in series with the gastrocnemius are in direct conflict with the benefits of impulsive leg extension and the vaulting inverted pendulum gait. This is one demonstration of the inherent conflict between mechanical and physiological optimality: mechanically, efficient terrestrial gaits with limbs are impulsive, but muscle efficiency (not to mention the rest of the body [41]) precludes exceedingly high forces.

## 3. Discussion

### 3.1. Distribution of human-like walking foot function

Any inference of function from a single evolutionary origin must be treated with caution. However, the mechanical form of the human foot, based on the interpretation of function presented here, suggests adaptation for economical walking from both the whole body mechanics and muscle physiology perspectives. This economical form of walking may have arisen in the hominin lineage as far back as *Australopithecus afarensis* (approx. 3.6 Ma). This is supported by the presence of a transverse and longitudinal arch [42] and evidence of a fully extended bipedal gait [43], together with early stance heel-strike [4,44] in *A. afarensis.* The contrast provided by chimpanzee feet and gait is striking, and the mechanical significance, to some extent, long been recognized: while they show the heel–sole–toe plantigrade stance, they completely lack the longitudinal arch [45,46] that stiffens the human foot, and ‘the digits do not provide an effective ‘toe snap’ of the sort that is significant in ‘smoothing’ the human stride at the end of the stance phase’ [47, p. 216].

Other large bipeds—for instance, ostriches (figure 1*e*) —do not have ground-striking ‘heels’ (at least during walking) projecting behind the functional ankle (in this case the tarsometatarso-phalangeal joint). Instead, they have extensive tendons acting as near-obligate distal springs. These presumably offer some advantage in terms of economy at high speeds, or gait robustness enabling rapid locomotion over uneven terrain. From ‘ankle’ structure and walking gait technique, therefore, humans appear more specialized for walking, and ostriches for running—not a surprising observation given a comparison of their top speeds.

Quadrupeds, even those that can be viewed as walking specialists, do not need the human foot design to achieve the same mechanical principles: a torque about the hip with appropriate phasing in the stride cycle enables suitable impulses to power walking economically [48], and the torquing muscles need not be loaded by body weight throughout the rest of stance [49]. Therefore, quadrupeds need not have large feet to walk economically.

### 3.2. Prosthetics and bipedal robot design

Non-muscle actuators, such as those used by robots or in powered prosthetics, often have a near-negligible energetic cost of isometric force. If such motors are used, there is no energetic benefit from having a human-like foot. In this case, more proximal actuators would have the advantage of moving the mass distribution proximally. This would enable relatively fast protraction (‘swing’) despite potentially compromised proximal musculature, both by reducing the natural pendular period of the leg, and the energy required to force the leg at above-pendular speeds. Thus, less human-like feet would allow the benefits of more natural human walking, in which steps are taken relatively quickly [50] allowing relatively short steps and high-walking speeds [11].
